# Optical-Theorem-Based Holography for Target Detection and Tracking

**DOI:** 10.3390/s25072203

**Published:** 2025-03-31

**Authors:** Mohammadrasoul Taghavi, Edwin A. Marengo

**Affiliations:** Department of Electrical and Computer Engineering, Northeastern University, Boston, MA 02115, USA

**Keywords:** holography, aerosol detection, optical-theorem

## Abstract

The development of robust, real-time optical methods for the detection and tracking of particles in complex, multiple-scattering media is a problem of practical importance in a number of fields, including environmental monitoring, air quality assessment, and homeland security. In this paper, we develop a holographic, optical-theorem-based method for the detection of particles embedded in complex environments where wavefronts undergo strong multiple scattering. The proposed methodology is adaptive to a complex medium, which is integral to the sensing apparatus and thereby enables constant monitoring through progressive adaptation. This feature, along with the holographic nature of the developed approach, also renders (as a byproduct) real-time imaging capabilities for the continuous tracking of particles traversing the region under surveillance. In addition, the proposed methodology also enables the development of customized sensors that leverage a controllable complex multiple-scattering medium and the derived holographic sensing technology for real-time particle detection and tracking. We demonstrate, with the help of realistic computer simulations, holographic techniques capable of detecting and tracking small particles under such conditions and analyze the role of multiple scattering in enhancing detection performance. Potential applications include the identification of aerosolized biological substances, which is critical for biosecurity, and the rapid detection of hazardous airborne particles in confined or densely populated areas.

## 1. Introduction

The ability to accurately detect and characterize particles is crucial in a wide range of scientific and practical applications related to environmental monitoring, air quality assessment, and national security [1,2,3]. In this context, optical-sensing methods are popular as they enable real-time and in situ examination of the nanoparticles present in typical media, such as aerosols [4,5,6]. Scattering-based techniques are quite effective in determining key attributes such as particle shape, size, and composition [7,8,9,10]. Some recent studies have introduced methods for designing sensors that measure extinct power in diverse environments for arbitrary fields using time-reversal mirrors or cavities [11,12,13,14]. However, these approaches depend on phase information, making them less suitable for optical and quantum systems, where only field intensities are measured. On the other hand, the same principle can be employed in the optical regime through holographic techniques. Berg et al. proposed and experimentally verified the possibility of measuring the extinction cross-section of spherical and non-spherical particles [15,16] under plane wave incidence. A related work is that of Ravasio et al. [17], where the digital holographic approach was adopted to simultaneously measure the optical extinction cross-section and morphological properties, such as cross-sectional shape and size, of single mineral dust particles. In another related work, Berg et al. introduced a simplified bi-telecentric lens system for digital in-line holography, enabling high-resolution imaging of free-flowing aerosol particles at the sub-micrometer scale [18]. In this work, we consider the possibility of generalizing some of these prior techniques so as to make them also applicable to complex, possibly random media, whereby the probing fields adopted in the illumination of the particles or targets are more arbitrary. For this purpose, we borrow from prior formulations of the optical-theorem (OT) in complex media [11,12,13] and combine them with the holographic sensing methods in [19] for the development of new OT holography, the objective of which is the remote sensing of the extinction or cross-section of a target embedded in a complex medium. This fundamental measure is then utilized as an indicator of target presence, where large values of the measured cross-section over a suitably selected threshold are indicative of target presence.

**Figure 1:**
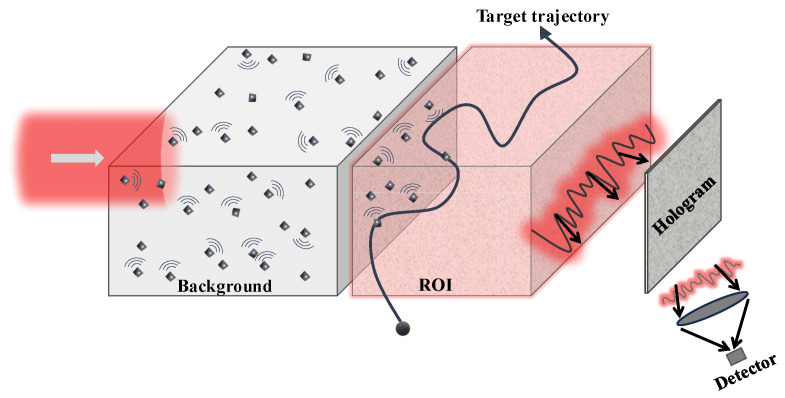
Conceptualization of the proposed holographic imaging system for the detection and tracking of targets traversing the ROI.

Figure 1 provides a schematic of the envisioned system. A laser beam is employed for the interrogation of a complex medium, which we assume to be composed of many randomly positioned scatterers with rather arbitrary scattering properties. The resulting perturbed waveform is adopted to probe the region under surveillance or region of interest (ROI), as shown in the figure, for the purpose of detecting and tracking particle motions in that region. The gathered data are captured holographically at the output, rendering a single-pixel or bucket detector measurement that is representative of the extinct power or energy of the scattering phenomenon. This quantity is adopted for the purpose of particle detection, and position-dependent variations of the associated response are adopted for the envisioned tracking. Of particular interest in this work is the holographic measurement of the particle’s extinction cross-section, with particular interest given to the situation where a particle is embedded in complex, possibly random media with multiple scattering interactions involved so that the corresponding extinction’s cross-section differs from the canonical value in free space. The sought-after sensing of the particle cross-section in a complex medium relies on a fundamental principle in scattering theory termed “the optical-theorem” [20]. Two application contexts are considered. First, in many practical scenarios, the sought-after particle detection and tracking needs to be implemented in conditions where the surrounding environment is composed of many multiple-scattering constituents. The complex medium scrambles the wavefronts, yielding complex waves. This makes detection and imaging quite challenging in such media. On the other hand, the same complex multiple interactions also enhance the degree of interactivity with the target. This may potentially enhance, in principle, the detectability of small particles relative to simpler media, e.g., under free space or weakly interacting environments. Thus, the first objective is to demonstrate holographic methods that are feasible for the detection and tracking of small particles in such media. In addition, we also show ways in which the complex medium does, indeed, enhance detectability. The second objective is to examine the possibility of developing sensors that are based on the use of a complex, multiple-scattering medium, along with holographic sensing equipment, for the detection and tracking of particles. The tracking function is achieved via holographically enabled backpropagation in a complex medium; this can be carried out either analogically, in situ, with the physical presence of the complex medium, or synthetically in computational form via the use of the associated differential hologram corresponding to the difference in the captured holograms of the scene with and without the intruding scatterer or particle. More importantly, the holographic method introduced by Berg et al. [15] focuses on the important case of plane wave excitation in free space. Our method is equivalent to the method in [15] for that particular special case. However, our emphasis in this work is on the case of non-plane wave excitation, where the particle is excited by a more complex wave coming from a complex embedding medium or background. Indeed, the proposed approach is based on the holographic sensing of the incident field associated with the complex background, as well as the total response, including the target in said background. The extinction measured in this approach is not necessarily equal to that in free space. Thus, in that sense, these developments extend the holographic extinction measurements in prior work to cases where one is interested in measuring the extinction of the particle in more complex surroundings. In view of the multiple-scattering interactions of the target and the medium, this cross-section can exhibit complex variations that are not present in free space, and, as will be indicated, this may have some interesting applications.

The remainder of the paper is organized as follows. In Section 2, we outline the envisioned operational concept and proposed methodology for optical-theorem-based holographic detection and tracking of small particles in complex media. Section 3 provides computer simulations that demonstrate the proposed technique’s effectiveness and advantages relative to alternative methods, such as non-holographic detection methods. The results in Section 3 are based on rigorous computations, including all the relevant multiple-scattering interactions in the complex medium. Section 4 provides concluding remarks.

## 2. Optical-Theorem-Based Holographic Detection

**Figure 2:**
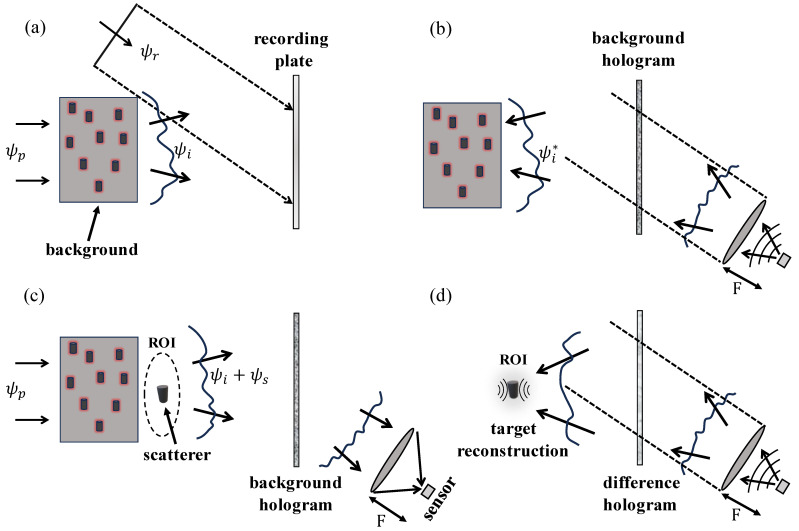
OT detector based on Leith-Upatnieks holography. (**a**) Background medium hologram. (**b**) Synthesis of the complex conjugate background field. (**c**) OT sensing in the presence of a target. (**d**) Backpropagation imaging using the difference hologram.

Consider a complex scattering medium, which may be composed, e.g., of a collection of randomly positioned multiple interacting scatterers. When this medium is illuminated with a probing wave $\psi_{p}\left(r\right)$, where r denotes position, then secondary sources are induced in the medium, which gives rise to scattering. The total field in the medium, which we term “background field”, is then given by

(1) $$\psi_{i}\left(r\right)=\psi_{p}\left(r\right)+\psi_{c}\left(r\right)$$

where $\psi_{c}\left(r\right)$ represents the scattered field due to the background alone. This is illustrated in Figure 2a. As shown in Figure 2c, if a scattering object or particle subsequently appears in this region, it launches further perturbative fields, henceforth to be denoted as $\psi_{s}\left(r\right)$ (the “scattered field”), in response to the total illumination $\psi_{i}\left(r\right)$ (the “incident field”). Obviously, the magnitude of the scattered power can be measured by sensing the scattered fields in all directions. On the other hand, more realistically, one employs finite sensing apertures only, and thus the practical question is, up to what extent it is also experimentally viable to measure or estimate such scattering power from finite-size sensors or through sensing apertures with limited-view access to wave information related to the scene of interest? More generally, ideally, the sensing should address the entire extinction, accounting for both scattered power (into all directions) and losses or dissipation (inside the target or particle). The answer to this important question is provided by the OT, which provides the required fundamental principle for the remote sensing (at a realistic limited-view aperture) of the entire extinction. Importantly, the most general forms of the OT (see, e.g., [20] and the references therein) apply to both arbitrary incident fields and quite arbitrary lossless media. In particular, it is well known (see, e.g., [11] and references therein) that the extinction power $P_{e}$ is measurable from the scattered field $\psi_{s}$ in a spatially-limited aperture through sensing mode R(r); in particular,

(2) $$P_{e}=-Ck_{0}^{-1}ℑ\int drR\left(r\right)\psi_{s}\left(r\right)$$

where *C* is a constant, *ℑ* denotes the imaginary part, $k_{0}=\omega /c_{0}$ is the wavenumber in free space, where ω is the angular oscillation frequency, $c_{0}$ is the speed of light, and R(r) emits (in radiation, acting as a source in the same background medium) the complex conjugated (c.c.) version of the incident field, $\psi_{i}^{*}\left(r\right)$; in particular,

(3) $$\int dr^{\prime }R\left(r^{\prime }\right)G\left(r,r^{\prime }\right)=\psi_{i}^{*}\left(r\right)\;r\in \mathrm{ROI}$$

where $G(r,r^{\prime })$ is Green’s function in the background. Thus, the key is that this OT receiver (R(r)) must be designed such that it is capable of generating in emission the c.c. counterpart $\psi_{i}^{*}\left(r\right)$ of the incident field $\psi_{i}\left(r\right)$ in the ROI. It follows that, for the practical purposes of the sought-after optical realization, the only requirement is the realization of an imaging system wherein the probing source and the intensity-only receiver are strategically arranged such that if the receiver acts as an emitter, it can effectively synthesize, in the background, the complex conjugated version of the probing fields in the ROI where targets are expected to appear, for the purposes of detection. Interestingly, this can be achieved via classical lens-based imaging systems in combination with holographic techniques. Moreover, in the proposed methodology, the sought-after OT detection can be carried out using a single detector (e.g., a bucket detector or single-pixel camera), which is a desirable feature for practical implementations.

Figure 2 illustrates the different steps of the required optical-theorem-based sensing. The system is designed to perform two functions: (1) the detection of particles based on the values of the extinct power $P_{e}$, where large values over a threshold indicate target presence; (2) the exploitation of the holographically captured data for the purposes of developing images that enable particle tracking. Moreover, as shown in the computer simulations, it is also possible to implement certain qualitative forms of tracking via the use of OT data only. The first step is the realization of an optical system capable of producing, in emission, the c.c. version of the probing fields due to the illumination of the complex background. Fortunately, holography provides a practical way to achieve this, particularly in the framework of the so-called Leith-Upatnieks holography [20], as we explain next.

As shown in Figure 2, the medium is illuminated with a probing wave $\psi_{p}\left(r\right)$, giving rise to the perturbed incident field $\psi_{i}\left(r\right)$ (comprising the probing wave ($\psi_{p}\left(r\right)$) plus the resulting background field ($\psi_{c}\left(r\right)$)), which is measured at the holographic plane with the help of a reference wave $\psi_{r}\left(r\right)$. The field at the hologram plane is equal to $\psi_{i}+\psi_{r}$, and consequently, the field intensity $I_{c}$ at point (x,y) in the hologram is given by

(4) $$\begin{array}{ccc} I_{c}\left(x,y\right) & = & |\psi_{i}\left(x,y\right)+\psi_{r}{\left(x,y\right)|}^{2}\\ & = & |\psi_{i}{\left(x,y\right)|}^{2}+\psi_{i}\left(x,y\right)\psi_{r}^{*}\left(x,y\right)+\psi_{i}^{*}\left(x,y\right)\psi_{r}\left(x,y\right)+{\left|\psi_{r}\left(x,y\right)\right|}^{2}. \end{array}$$

This enables the recording of transparency t(x,y) at the hologram plane, which constitutes the recorded hologram; in particular, it is proportional to $I_{c}$:

(5) $$t\left(x,y\right)=BI_{c}\left(x,y\right)$$

where *B* is a constant. When this holographic transparency is excited with the c.c. form of the reference wave, $\psi_{r}^{*}\left(r\right)$, this gives rise to an induced source at the hologram plane; in particular,

(6) $$\rho \left(x,y\right)=t\left(x,y\right)\psi_{r}^{*}\left(x,y\right)$$

the generated fields of which have, according to (5), four components. It is well known that these components can be suitably isolated if the reference wave’s angle of arrival relative to the information-carrying beam ($\psi_{i}\left(r\right)$) is properly chosen, e.g., for plane wave reference waves; this is achieved via the classical Leith-Upatnieks approach. Now, assuming that the holographic system has been designed in this way, then it follows that the induced source in (6) generates the c.c. field $\psi_{i}^{*}\left(r\right)$ in the ROI, as desired. This principle is illustrated in part (b) of Figure 2, where a point source located at the bucket detector acts as an emitter so as to launch in the presence of the focusing lens the c.c. form of the reference wave. Assuming that the reference wave is a plane wave, as shown in the figure, then the required source (for the synthesis of the c.c. reference beam) can be created by placing the point source at the focal plane of the lens, as illustrated in Figure 2b. When this c.c. reference wave impinges on the hologram, it gives rise to the emission, into the ROI, of the sought-after c.c. form of the incident field, as required for optical-theorem-based sensing. Thus, Figure 2a,b illustrate the procedure for the required data-driven design of a sensor capable of generating in its complementary radiation or emission counterpart the c.c. form of the incident fields in the ROI. This is carried out in two steps. First, the incident field is recorded holographically at the hologram plane, with the help of a reference wave, e.g., the plane wave shown in Figure 2a. The sensor is placed such that when it acts as an emitter in the corresponding lens-based imaging system (where the sensor is placed at the focal plane of the lens, as shown in the figure), it launches the c.c. form of the reference wave. Once this is achieved, then the sensor has the capacity to function as an OT detector for the measurement of the extinct power, as we explain next.

When a scatterer, such as a particle, appears in the ROI, then this causes a perturbation to the bucket detector’s intensity. Like the construction procedure for the OT detector, the measurement stage also involves two steps. First, we measure (at the OT bucket detector) the intensity of the corresponding field, which is denoted as $I_{b}$. It is given by

(7) $$I_{b}\left(r_{0}\right)={\left|\psi_{b}\left(r_{0}\right)\right|}^{2}$$

where $r_{0}$ denotes the detector’s position. This intensity corresponds to the background field alone. If a target appears in the region, then the total field $\psi_{t}\left(r_{0}\right)$ arriving at the detector changes and is now given by the sum

(8) $$\psi_{t}\left(r_{0}\right)=\psi_{b}\left(r_{0}\right)+\Delta \psi \left(r_{0}\right)$$

where $\Delta \psi (r_{0})$ denotes the perturbation field arising from the presence in the ROI of the target. In view of (8), it follows that the intensity at the detector becomes

(9) $$I_{b+s}\left(r_{0}\right)=I_{b}\left(r_{0}\right)+2ℜ\left[\psi_{b}^{*}\left(r_{0}\right)\Delta \psi \left(r_{0}\right)\right]+{\left|\Delta \psi \left(r_{0}\right)\right|}^{2}.$$

It follows from (8) and (9) that if the perturbation field is relatively weak relative to the background field, in particular, $|\Delta \psi \left(r_{0}\right)\left|<<\right|\psi_{b}\left(r_{0}\right)|$, an expected condition for small targets is then

(10) $$\Delta I=I_{b+s}-I_{b}≃2ℜ\left[\psi_{b}^{*}\left(r_{0}\right)\Delta \psi \left(r_{0}\right)\right].$$

Now, it can be shown (see, e.g., [21], Equation (30), and [11], Equations (32) and (33)) that the quantity ΔI in (10) is proportional to $P_{e}$ in (2), i.e., ΔI is, apart from a multiplicative factor, equal to the sought-after extinction power $P_{e}$, as desired. This completes the derivation of the optical-theorem-based holographic detector of the extinction power. In addition, if the system incorporates a second hologram in the presence of the target, then it becomes possible to also achieve backpropagation-based imaging in this scene via excitation at the sensor’s position and the adoption of a difference hologram, given by the difference in the second and first holograms. Moreover, if the system implements continuous recording of the background hologram, then, for constant updating, an additional capability emerges: continuous re-imaging of the scene, which focuses via standard backpropagation into the target region, thereby rendering extra target tracking capabilities.

## 3. Computer Simulations

We illustrate the application of the developed optical-theorem-based holographic system for the detection of scatterers passing by the ROI, which is located near a complex scattering medium. The assumed medium complexity is, in general, detrimental in a number of tasks based on conventional imaging. However, the same complexity, which generally involves strong multiple-scattering or multipath interactions, also brings a high degree of wave interactivity with the target. For a suitably designed system, this feature can enhance target detectability, as desired. Thus, small cross-section targets that may be hard to detect in free space or other simple background media become highly detectable with this system, thanks to the embedding, within the system, of the corresponding complex, multiple-scattering material. Thus, the complex medium is integral to the sensing apparatus. Moreover, even though the focus in the following is the situation of an invariant background, the same approach is also applicable to more general, varying media, in which case data updates need to be constantly incorporated into the detection process, of course. This concept is implicit in some of the illustrations pertinent to tracking, where it is assumed that the in situ background holograms are captured continuously.

In the simulations, electromagnetic scattering is modeled in two-dimensional (2D) space for a complex medium consisting of a collection of randomly distributed, parallel dielectric cylinders, the multiple-scattering interactions of which are fully incorporated via standard methods (see, e.g., [22,23], and the references therein). Of particular interest is the detection of a target or “particle” crossing the vicinity of the complex medium. The problem of detecting two or more targets (e.g., a stream of particles) is also examined, and the unique response characteristics of different random sets of cylinders are also briefly discussed. In our numerical calculations, the wavelength (λ) serves as the reference for normalizing both the simulation domain and particle dimensions. As a result, our proposed techniques are adaptable to any arbitrary wavelength. However, the primary applications we consider fall within the visible and near-infrared spectrum, where, typically, only intensity data are available. Regarding the scattering regime, Rayleigh resonance occurs when the particle size is much smaller than the wavelength, which is not the case in our simulations [24]. Based on the diameter of the dielectric cylinders used, the observed scattering behavior corresponds to the Mie regime. The impact of these two scattering types on holographic recording and particle detection is significant. Rayleigh scattering is isotropic, leading to low-contrast speckle patterns, making it challenging to resolve individual particles. In contrast, Mie scattering is anisotropic, which is characterized by strong forward scattering. Nevertheless, increased scattering enhances speckle contrast, which can be leveraged for improved phase retrieval. This, in turn, leads to more precise particle detection and tracking.

Electromagnetic scattering involving multiple objects is quite challenging due to the need to account for the coupling between the elements. To satisfy the relevant boundary conditions on the surface of the cylinders, we made use of the well-known translational addition theorem. The latter allows the representation of the scattered fields from one cylinder to be incident fields on another one, facilitating the use of a common reference origin for both. The literature is extensive, and numerous studies have focused on calculating scattering from cylindrical scatterers, as well as on analyzing multiple scattering interactions within groups of such scatterers [25,26]. A convenient way to model this problem is to use transverse magnetic (TM) and transverse electric (TE) scalar potentials that can be calculated analytically for a large number of scatterers [23,27]. In particular, we considered a TM incident wave ($H_{z}=0$) of the form

(11) $$\begin{array}{c} u_{j}^{i}=-\frac{1}{k}\sum_{n=-\infty }^{\infty }i^{n+1}\left[J_{n}\left(\rho_{j}\right)\epsilon_{j}\right]\exp\left(-in\gamma_{j}\right) \end{array}$$

where $\left(r_{j},\gamma_{j}\right)$ are the polar coordinates with respect to the origin of *j*-th scatterer, $\rho_{j}=kr_{j}$, $J_{n}\left(\;\cdot \;\right)$ is the Bessel function of order *n*, and $\epsilon_{j}=\exp\left(id_{1j}k\cos\left(\beta_{1j}\right)\right)$ is the phase-shift factor for the incident wave, where $d_{1j}$ is the separation between the first cylinder and the *j*-th one. Moreover, $\beta_{1j}$ is the angle that the line connecting the centers of the first and *j*-th cylinders makes with the *x*-axis. To continue, one needs to write the electromagnetic boundary conditions for every one of the scatterers. This gives rise to suitable continuity conditions that *u* and the normal derivative ∂u/∂r must obey. As mentioned, the translation addition theorem facilitates this process. The scattered fields in the exterior part of the cylinders can be written as

(12) ujs=1k∑n=−∞∞in+1[Hn(ρj)]exp(−inγj)bnj

where bnj represents the corrected scattering coefficients that are obtained from the boundary condition at $r_{j}=a_{j}$. Additionally, $H_{n}\left(\;\cdot \;\right)$ is the Hankel function. It should be noted that in the absence of multiple scattering (i.e., for a single cylinder), the single-scattering coefficients are already known, of course [28].

Furthermore, the TM potential in the immediate vicinity of cylinder $C_{j}$ consists of three components, including the primary and secondary incident and scattered waves. If proper boundary conditions are applied to these equations, then the corrected scattering coefficients can be computed as

(13) bnl=bnl(0)[ϵl+in+1∑j=1j≠lNexp(inβlj)jlB−n]Bnlj=∑s=−∞∞is+1Ψljexp(−isβlj)Hn+s(δlj)bsl

where $B_{n}$ is the coupling term, and $\Psi_{lj}={\left(-1\right)}^{s}$ if l>j and ${\left(-1\right)}^{n}$ otherwise. These two equations are coupled and can be solved either iteratively or using inverse methods depending on the separation between the cylinders. The most accurate solution is obtained through the inverse method, where the mentioned coupled equations should be written in a matrix form:

(14) $$M={\left(I-C\right)}^{-1}S$$

where *S* and *M* denote vectors of single and multiple scattering coefficients. *C* is the coupling matrix that contains the coupling terms between single and corrected scattering coefficients in Equation (13). By solving this matrix inverse equation, the corrected scattering coefficients are obtained, which completes the parameters required for finding the scattered fields in the simulation region.

**Figure 3:**
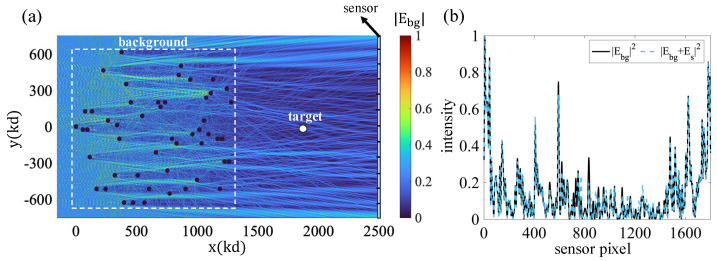
(**a**) Background medium and the corresponding field intensity. (**b**) Background field intensity and total (background plus scattered field) intensity at the hologram aperture.

Figure 3a shows the geometry considered in the simulations. It illustrates the background medium, which is composed of a cluster of cylinders with random positions and refractive indices 3<n<4 with a radius of $r_{s}=3\lambda$. The scatterers are placed in the highlighted rectangular region, which has dimensions of $0<l_{x}<400\pi \lambda$ and $-200\pi \lambda <l_{y}<200\pi \lambda$. The ROI is assumed to be to the right of the scattering region, as shown in the figure. As shown in the figure, the intensity of the corresponding background field in that region is quite complex, as is expected from the adopted medium’s complexity. Part (b) of the figure shows the field intensity profile at the hologram aperture for both the background field and the total (background plus scattered field). The target consists of a single cylinder with a refractive index and radius of n=1.2 and $r_{s}=1.5\lambda$, respectively. The two plots are very similar for the target considered. This shows that, at least for weak scattering targets, it is very difficult to detect the target presence based on intensity-only measurements at the hologram plane. In contrast, the OT method adopted in this work is a coherent detection method and, as we show next, it reveals both the target presence and the unique features that can be associated with the target’s trajectory.

**Figure 4:**
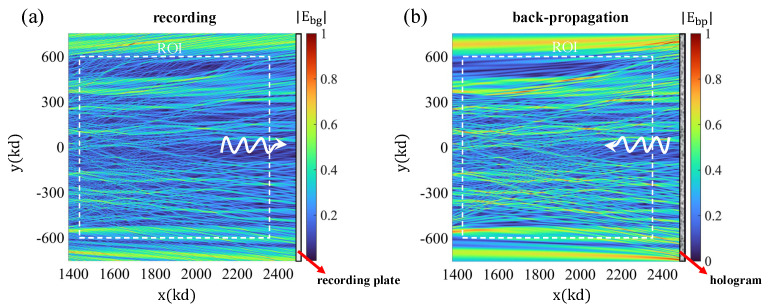
(**a**) Recording stage of hologram generation. (**b**) Background field reconstruction in the ROI when the bucket detector acts as a source.

The effectiveness of the OT approach as a method to detect the scattering cross-section rests on the ability of the sensor system to radiate (in the complementary transmission mode) the c.c. version of the probing field adopted to interrogate the target. The key attribute is the system’s ability to reproduce, in transmission, the c.c. field, at least within the ROI where targets are expected to appear. Figure 4 demonstrates the viability of the system in Figure 3 for the effective synthesis of the c.c. probing field in the desired ROI. Figure 4a shows a plot of the intensity of the incident wave probing the ROI. The figure shows the hologram plane, where the field is recorded holographically with the help of a reference wave. Part (b) of the figure shows the corresponding backpropagation reconstruction, corresponding to the synthesis of the c.c. field using the same hologram recorded in part (a) upon excitation with the relevant c.c. form of the reference wave. This is achieved by launching a probing field from the single-pixel or bucket detector region, which, upon interacting with the lens, gives rise to the c.c. form of the reference wave adopted in the hologram generation step. Comparing the two plots of the field intensity, we conclude that they are very similar, as desired. Thus, the required c.c. field reconstruction is successful with this sensing apparatus, as required for the applicability of the OT sensing approach.

**Figure 5:**
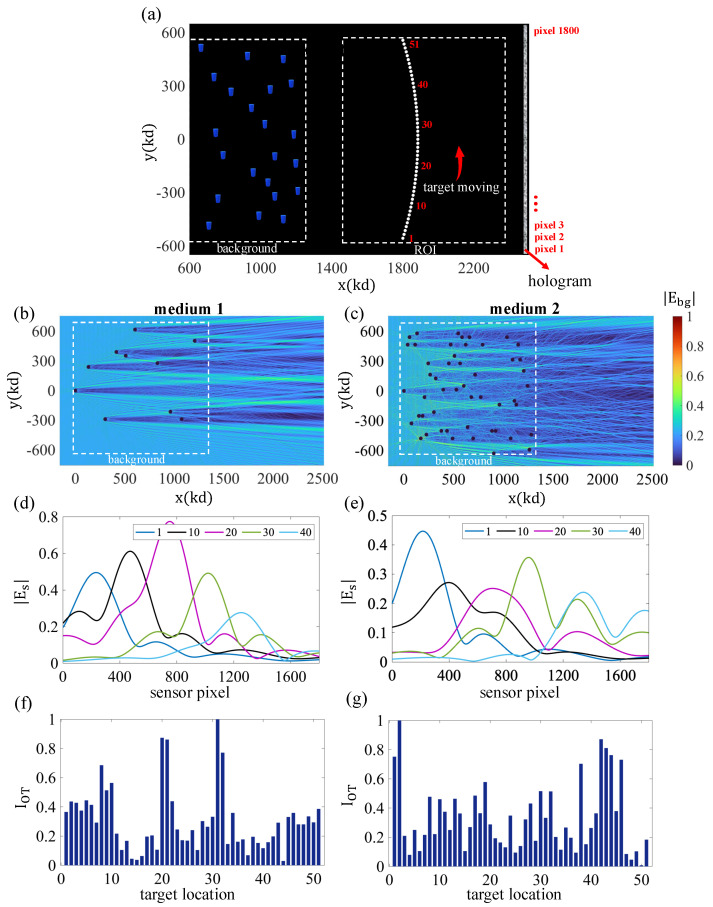
Intrusion scenario, where a single cylindrical particle enters the ROI and traverses a radial path. (**a**) Two random background media with different scatterer densities are utilized in this demonstration, which are called medium 1 (eleven scatterers) and 2 (51 scatterers). (**b**,**c**) Normalized intensity of the total field that the background media generate throughout the ROI. (**d**,**e**) Intensity of the scattered fields from the moving target present in selected locations on the hologram aperture. (**f**,**g**) Distribution of the normalized value of the OT detector when mediums 1 and 2 are used, respectively.

Another feature of the developed method is that it also allows inferences about the target’s location and trajectory as it moves in the region. As an example, we have considered a scenario where the cylindrical particle (n=1.2, $r_{s}=1.5\lambda$) moves in a radial path (r=600πλ, −0.3<θ<0.3 (radians)). The simulation geometry is illustrated in Figure 5a. We examined the tracking capabilities of the system using different methods. First, we discuss the role of medium complexity in both detection and tracking using the OT approach only. For this purpose, we compared two media with different numbers of scatterers. Parts (b) and (c) of Figure 5 depict the configurations of (1) background medium 1, which contains 10 scatterers, and (2) background medium 2, which contains 50 scatterers. In these experiments, we considered refractive indices in the range of 3<n<4. The positions were selected randomly to simulate complex random media. As can be seen in these figures, the medium with more scatterers generates a more complex probing field in the ROI, as anticipated. In addition, Figure 5d,e show the scattered fields corresponding to different target positions. Given the size and shape of the scatterer adopted in the experiments, it is expected that it should have a relatively symmetric scattering pattern in the forward direction. This is, indeed, consistent with the results in Figure 5d,e. The OT results are shown in Figure 5f,g. The OT indicator is normalized in these plots to enhance interpretation.

The pattern of the computed OT indicator in the case of medium 2 clearly shows more abrupt variations as a function of target position. Thus, increased medium complexity translates to greater variability in the measured OT response. This could be useful in detecting the nanoscale movement of the particles, as even a tiny variation in position gives rise to a noticeable change in the OT indicator. In addition, if the adopted medium’s OT response is stored, this could be used as a template for the estimation of the target position, e.g., the peaks in the OT plots can be associated with the maxima of the stored background field to detect the proximity of the target to given reference points, as desired. Next, we discuss another tracking technique that relies on the difference between holograms based on two background holograms captured at different times. These can be consecutive times, or we can use the background hologram of a prior past time as the reference for the image generation process.

**Figure 6:**
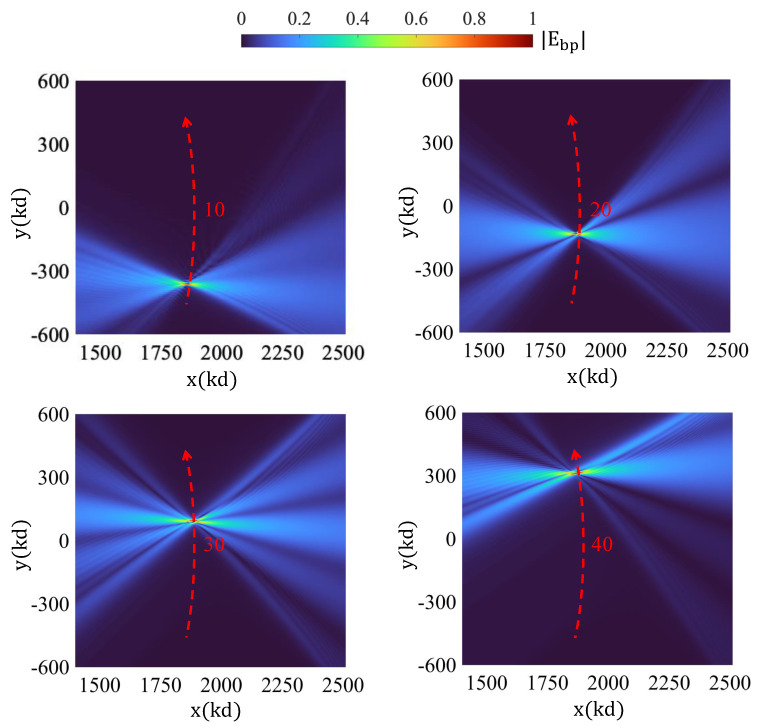
Tracking of the particle via backpropagation-based imaging based on the difference hologram.

Figure 6 shows the backpropagation-based imaging based on the difference hologram corresponding to two different times. This can be, e.g., the reference background hologram corresponding to the medium, plus an additional hologram corresponding to the data-gathering steps, where one can measure not only the OT sensor intensity but also capture a second hologram pertinent to the scene at the same time interval. The latter hologram includes the effect of both the background and the target, and therefore, the subtraction of the two holograms, which we term “difference hologram”; this contains information about the target’s whereabouts. Under point-source-like excitation at the bucket detector, it is possible to generate a real-image reconstruction of the target in the ROI, and this can be carried out, e.g., with a tandem holographic system, implementing the complementary function of tracking. Alternatively, this can be carried out computationally from said difference hologram via conventional backpropagation from the hologram aperture. The backpropagation images shown in the figure correspond to field intensity and are quite impressive; they consistently focus on the correct target position as it trespasses the region along the trajectory shown.

**Figure 7:**
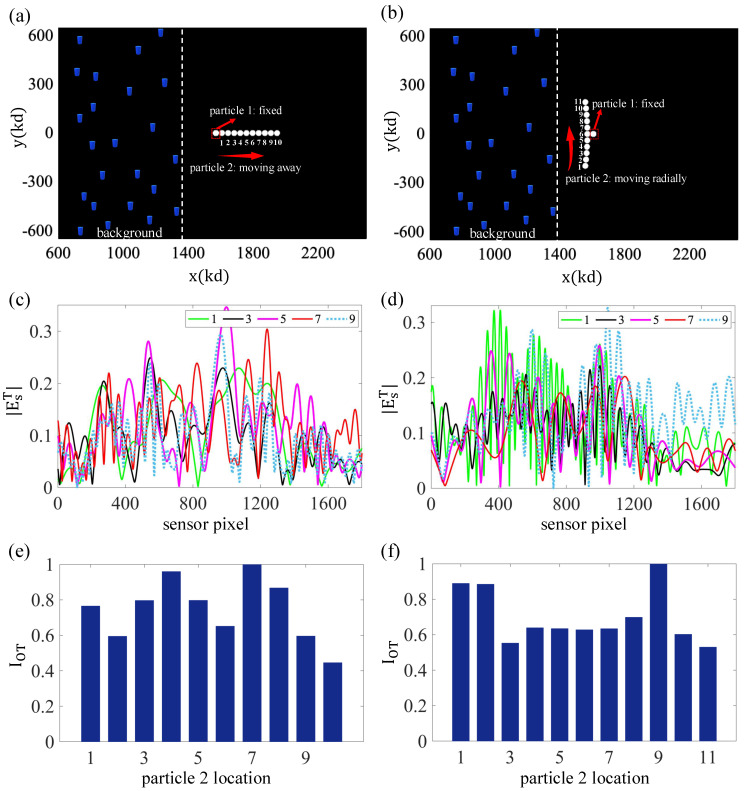
Analyzing the effect of relative movements of two already present cylindrical particles on the observed OT value. (**a**,**b**) Configuration adopted in the experiments. One of the particles is kept at a fixed location. The second particle moves in the trajectory shown. (**c**,**d**) Intensity of the total scattered field from both particles in selected locations of the moving particle. As can be seen, this total scattered field $E_{s}^{T}$ is highly dependent on the relative positions of the particles, mainly due to multiple-scattering effects between them. (**e**,**f**) OT indicator versus target position.

As a final OT analysis, in Figure 7, we considered a scenario that further illustrates additional features of the entire approach. In this case, we considered two targets in two different trajectories: horizontal motion in part (a) of the figure and vertical movement in the companion part (b). One of the particles remains fixed, and the other particle moves in the shown trajectory. As the plots reveal, the scattered field changes a lot from one sense of motion to the other. The OT indicator also exhibits noticeable differences in these two trajectories. Motivation was provided by a possible “target separation problem”, where a target may appear, effectively, as a single entity (initially), as depicted in part (a) of the figure, but subsequently becomes separated into two different targets. As the figure reveals, the OT responses of the two possible trajectories are quite distinguishable, and, clearly, they can, in principle, be discerned via the OT indicator if prior stored information is available. In addition, we also ran additional simulations (results not shown) of the associated backpropagation imaging (as in Figure 6), and we found that the targets became clearly differentiated in the images, as desired.

Our simulations model the background medium, which involves considering the multiple scattering between elements (dielectric cylinders in this case) and also target scattering in the presence of that background field. As a proof of principle, we have performed OT integration on the hologram aperture, which is equivalent to performing it on the lens aperture. Due to the nature of our work, we have considered certain idealized parameters in our model, such as coherent illumination of the light source, the assumption of a stable scattering medium, an idealized lens, and, most importantly, a noise-free bucket detector. In a practical setting, the presence of different types of noise in extinction measurements can be concerning [29,30]. The proposed holographic architecture consists of a bucket detector (single-pixel camera), where shot noise, which follows Poisson statistics due to the quantum nature of light, as well as thermal noise and other detector-related noises, are crucial to consider [31,32,33]. In the presence of noise, extinction measurements can become unreliable, especially when the target nanoparticle field is weak (low-light conditions). Fortunately, there are various methods to mitigate the effects of detector noise, such as increasing the optical power of the illumination source to improve the signal-to-noise ratio (SNR). Longer integration times can also be used to average out noise fluctuations, although this may introduce thermal noise considerations. Nevertheless, other computational methods, such as background subtraction, can also be helpful in suppressing static noise sources [34,35,36].

**Figure 8:**
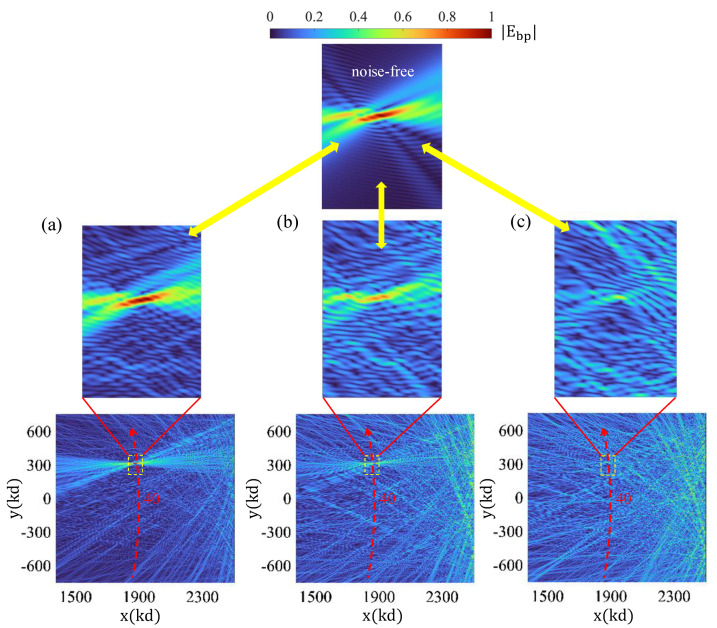
Effect of noise on particle tracking performance.

To conclude this section, we briefly discuss some of the challenges associated with the experimental implementation of the holographic-based particle detection and tracking methodology. Generally, in holographic systems, multiple sources of noise can degrade system performance, including speckle noise and unwanted background scattering. Moreover, speckle noise is a fundamental challenge in holography, arising due to the coherent nature of the light source, which causes unwanted interference patterns in the recorded images. Various methods exist for reducing speckle noise [37,38,39]. For instance, spatial filtering can be employed to reduce high-frequency noise components that contribute to speckle [40]. If speckle noise exhibits polarization dependence, a polarization filter can be used to suppress the unwanted speckle contribution. Additionally, temporal or angular averaging methods can be effective in mitigating noise effects in the bucket detector. Furthermore, adjusting the coherence properties of the illumination source can significantly suppress speckle noise. These methods include using a laser with a shorter coherence length, as an example [41,42,43]. To analyze the impact of noise in our holographic system, we consider two primary noise sources: speckle (multiplicative) and additive noise. Moreover, multiplicative noise is modeled as

(15) $${\tilde{H}}_{diff}=H_{diff}\times A_{\mathrm{speckle}}\times e^{i\phi_{\mathrm{speckle}}}$$

where $H_{diff}$ and ${\tilde{H}}_{diff}$ are the noise-free and noisy difference holograms, respectively. The random variations in amplitude $A_{\mathrm{speckle}}$ follow a Gaussian (normal) distribution, with a mean value of 1 and variance of $\sigma_{\mathrm{speckle}}^{2}$ to ensure that the overall signal intensity remains stable. Moreover, the random phase shifts are assumed to be uniformly distributed within a given range [−π/2,π/2]. In the simulation, additive noise is introduced as a complex Gaussian random variable to model detector noise and background fluctuations as

(16) $${\tilde{H}}_{diff}=H_{diff}+n_{\mathrm{add}}$$

where the noise term $n_{add}$ is generated as a sum of independent, normally distributed real and imaginary components, each scaled by a noise factor $\sigma_{A}$. In Figure 8, we consider the previously identified difference hologram corresponding to the 40th particle position. We comparatively examine the effect of three different noise signals. Parts (a), (b), and (c) of Figure 8 show the backpropagation results obtained by assuming noise factors of $\sigma_{A}=0.1,0.25,0.5$, respectively. In these simulations, we considered total noise as a combination of a multiplicative component, with $\sigma_{speckle}=0.1$ and an additive part. It is noteworthy that the mean value of the original difference hologram signal was computed as $M_{\psi_{s}}$ = 0.0725. The obtained reconstruction images demonstrate the sensitivity of the signal to the addition of speckle and background noise. We have observed that the additive noise plays a critical role in the performance of the particle tracking, as it introduces fluctuations in the recorded intensity patterns, affecting the accuracy of backpropagation-based reconstructions and the OT indicator. Higher levels of additive noise degrade the SNR, making it more challenging to differentiate between the background medium and the tracked target, particularly for weakly scattering particles. Finally, the exploration of methods to enhance the operational performance of the proposed OT platforms will be left for future research.

## 4. Conclusions

In this work, we have developed an optical-theorem-inspired holographic method for the detection and tracking of particles embedded in complex multiple-scattering media. Motivation is provided by a number of applications, e.g., in the biomedical, environmental, and defense areas, where one seeks to detect and characterize targets of interest, such as particles, aerosols, etc., in real time and under evolving conditions, and where the embedding medium is typically complex and random. In this work, we have adopted the extinction cross-section as the key physical descriptor of the particle’s presence under the assumption that the sought-after minimally detectable target size or associated extinction is approximately known. This permits, in principle, the suitable calibration of the sensing apparatus through proper threshold level selection so as to render the desired probability of detection and false alarm under the anticipated conditions, e.g., trade-offs, risk levels, etc. The classical OT of scattering theory provides the underlying physical principle for the proposed detection system. We proposed the use of a holographic system, where the reference wave is an oblique-incidence plane wave arriving at the hologram plane. The well-known principles of Leith-Upatnieks holography are, therefore, applicable so as to enable the critical isolation of the four different components of the field when the hologram is illuminated either by the reference wave or the c.c. form of that wave for the purposes of image reconstruction. The detector itself is formed by a single-pixel camera or bucket detector positioned at the focal plane of the lens for the creation of a lens-law-obeying imaging system with the laser source that generates the reference wave. This ensures that the bucket detector emits, in radiation, the c.c. form of the reference wave, as required for the applicability of the OT. This enables, in turn, the launching of the c.c. form of the background medium field through the illumination of the “background medium hologram” with this c.c. reference wave, thereby completing the requirements that the sensor must obey in order to function as a detector of scattering extinction, as desired. The proposed detection and tracking methods have been illustrated numerically, incorporating all the pertinent multiple-scattering effects for background media composed of random collections of dielectric nanocylinders. We have shown that the OT approach enables the sought-after particle detection under conditions where the presence of the target is virtually invisible in the associated intensity-only image at the hologram plane. Moreover, although not shown in the paper, this signal-enhancing effect associated with the OT method is even more noticeable under noisy conditions since the OT approach is based on coherent processing, in which noise cancellation is very effective, as is well known. We have also studied the dependence of the calculated extinct power on target position, as well as the effects associated with movement direction and the role of additional targets in the region. The results indicate that the OT approach is also potentially applicable to the associated task of tracking. In addition, the obtained results also illustrated the use of the difference hologram, corresponding to successive hologram captures, for the backscattering-based imaging of the target, through which tracking can also be achieved. This can be carried out either computationally (synthetically) or through a companion (in tandem apparatus) for real-image reconstruction based on said stored holograms. The derived detector is single-pixel-based, and it also relies on the exploitation of the background medium itself. In that sense, it can be thought of as an in situ compressive detector; it measures extinction, a physical indicator of target presence, via a single, well-localized sensor that is embedded into (or is part of) the surrounding medium of interest. The data-driven nature of this sensing approach also makes it universally applicable, and, indeed, the sensor can be thought of as being adaptive to the medium. Moreover, the adaptability properties of the derived optical-theorem-based system also make it viable for real-time detection and tracking in dynamic, varying environments. In particular, in the derived approach, the incident field associated with the background is recorded holographically; this operation can be implemented constantly at regular intervals for continuously updating the associated optical-theorem holograms, through which the measurement of extinction becomes possible at the single-pixel sensor. This possible dynamicity of the hologram itself is also what enables the nonstop updating of the associated “difference hologram”, through which the imaging of the target becomes accessible despite the medium’s complexity and dynamic variability. Future directions include the possible application of the results derived in this paper to other fields, such as physical layer security, encryption in complex media, secure optical-theorem-based communications, and related areas. We are currently exploring these areas and plan to report on the associated research developments in the future. 

## Data Availability

The data presented in this study are available on request from the corresponding author.
